# Meetings of Societies

**Published:** 1949-04

**Authors:** 


### Meetings of Societies

### CLINICAL SOCIETY OF BATH

7th January: Mr. J. Sanson, F.R.C.S.; "**Selene and Periodicity**". The speaker exemplified the very widespread and varied forms of life, especially of the sea-urchin type, whose gonadal activity and spawning were markedly influenced by the phase of the moon. Lunar influences were recognized so far back as written and legendary history could be traced in the Mediterranean and among the primitive tribes of the South Seas. The latter had for centuries accurately anticipated the maturing of the palalo worm exactly one week after the August full moon and set aside this time of fabulous catches for fishing festivals and celebration. The belief that human fertility and menstruation were controlled by lunar sources was based largely on superstition, but it had been demonstrated that plant germination was positively influenced by moonlight. A possible explanation of this lay in the 15–25 per cent of polarized light in moonlight during certain phases only.

Mr. Sanson cited many other examples of moonlight activity, ranging from the migration of eels to the Sargasso sea to bathing parties on the Californian beaches in search of sea-urchins. He doubted whether any true effect was produced by the moon in cases of lunacy, or whether the reputed link-up between therapeutic medicine and the lunar phases was more than superstition.

4th February: Mr. A. E. Burton, F.R.C.S., read a paper on "**Rehabilitation in Peace and War**".

The paper stressed that, although industrial medical officers had worked on the immediate and somewhat short-term rehabilitation of the injured workman since 1930–35, it was only during and since the recent war that the long-term functional and psychological rehabilitation problems had been fully evaluated. This had been most fully worked out in the R.A.F. largely because of the ability to fly home injured personnel during early stages of the war and thus establish special units, and also because of the exceptional value both financially and otherwise of the trained aircrew personnel. Partly because of this, and their high morale, it was possible to return no fewer than 83 per cent. of injured aircrew to some form of flying activity.

In peace-time there existed an equal need for a fully thought-out treatment in adequate and pleasant centres, with proper regard to the easing of financial and psychological stress and imbuing a standard of high morale and an attitude of self-help in the injured workman. Liaison schemes between such centres and the Ministry of Labour were already in existence and were capable of a great deal of development, but Mr. Burton stressed very forcibly the need, not only for producing a functionally good result, but a fit, happy and willing human being.

4th March: Discussion on minor medicine and surgery.

Clinical cases were shown at all three meetings, perhaps the most interesting being two cases of total gastrectomy for cancer by Mr. Kindersley. A third case, with anastamosis of the œsophagus to the small intestine was shown by Mr. S. Glaser. Mr. Kindersley also showed a case of removal of the duodenum and head of the pancreas for neoplasm, the stump of the pancreas, bile duct and stomach being anastamosed independently into the small gut. Dr. H. Lovell Hoffman showed a case of tumour of the optic thalamus successfully treated by irradiation. Progress had been satisfactory and epileptiform fits were decreasing in frequency. The patient attended as an out-patient.

---

### CARDIFF MEDICAL SOCIETY

MEETINGS held, 11th November: Dr. Mary Cross, "**The Premature Infant**". 9th December: Mr. Dickson Wright, "**The Surgical Treatment of Hypertension**". 13th January: Sir Maurice Cassidy, "**The New Cardiology**". 10th February: Dr. Dillwyn Thomas, "**Surgery in the Treatment of Pulmonary Tuberculosis**".

---

### GLOUCESTER MEDICAL SOCIETY

*Meetings held.* The following B.M.A. meetings are being held during this Winter Session. 10th March: Dr. Erskine on "**Some Common Skin Disorders**".

*Forthcoming meetings.* 12th May: Annual Meeting. 9th June: Dr. C. E. Lakin, F.R.C.P., F.R.C.S., "**Some Enigmas of Diagnosis**".

### WEST SOMERSET MEDICAL CLUB

ON 17th February: Dr. Charles Wroth, D.M.R.E., at Bridgwater Hospital; "**X-ray Co-operation**".

Dr. Wroth emphasized the importance of consultants forming a close liaison with the radiologist, giving the radiologist a reasonable history of the case and allowing him to choose the "positions" which would help in the diagnosis.

"Too often, consultants, especially orthopædic surgeons, consider that radiographers have the power of telepathy. . . . Radiographers have a very concentrated technical training but, unfortunately, this does not include pathology; it would help considerably in many cases if the consultant could approach the radiologist directly." Dr. Wroth showed many interesting films to illustrate the numerous pitfalls of interpreting radiographs.

23rd February: Mr. N. P. Capener, F.R.C.S., at the Ministry of Pensions Hospital, Musgrove Park, "**Osteo-Arthritis of the Hip**". Mr. R. F. Winckworth, F.R.C.S., in the chair. Mr. Capener explained in great detail the condition of osteo-arthritis in the adult. The old operations and the more recent advances were explained and some excellent slides were shown. Several clinical cases from the Ministry of Pensions were exhibited and it was noted the remarkable recovery which many of these people had made.

---

### DEVON AND EXETER MEDICO-CHIRURGICAL SOCIETY.

ON January 20th Dr. J. Gibbens, M.R.C.P., gave an address on the evils of over-feeding young children, reported in the *Lancet*, February 5th, 1949.

---

### PLYMOUTH MEDICAL SOCIETY

PRESIDENT for 1948–9 Dr. H. Guy Ludolf; Secretaries Dr. S. M. Davidson and Dr. Robert Walker. The Library is to be installed in the Board Room of the Prince of Wales's Hospital where most of the meetings are held. The Annual Dinner and Dance were held at the Duke of Cornwall Hotel in October. 2nd November, clinical meeting: Mr. Riddell shewed a granulosa-celled tumour; Dr. Lister, a case of multiple myelomatosis; Mr. Duncan Wood, a case of plasmocytoma of the femur and Dr. Steed, a patient who having had gout, tabes and amœbic dysentery, with a calcified cyst in his liver, also had a liver cyst from which several pints of fluid were removed at intervals. 19th November: Professor Drew Smythe, gynæcological cancer, its early diagnosis and the place of radium in treatment. 10th December: films of anæsthesia and of patent ductus arteriosus. 14th January: Mr. Ashton Miller, genito-urinary carcinoma. 1st February, clinical meeting: Dr. Croft showed cases of acromegaly and Cushing's syndrome and discussed the pituitary disorders. Dr. Larks exhibited urethral calculi, Dr. Shore and Dr. Lister shewed a case and discussed the treatment of tuberculous meningitis by streptomycin. Mr. Wilson discussed the diagnosis and surgical treatment of hydronephrosis associated with aberrant renal artery. 25th February: Mr. Wilson, "**Lumps in the breast**". Dr. Croft, "**Rheumatism in the Home**"; the diagnosis, prevention and treatment of rheumatoid and osteo-arthritis, dealing mainly with aspects which affected the general practitioner.

A further clinical meeting takes place on 25th March and Professor Lambert Rogers will address a joint meeting with the B.M.A. on "**Sciatica**" on 11th March.

---

SOUTH-WESTERN LARYNGOLOGICAL ASSOCIATION

THE third meeting of the session was held at the Royal Victoria and West Hants Hospital, Bournemouth, on Saturday, April 2nd. Messrs. Cowan, Mackenzie Ross, Salkeld and Adeney showed twenty extremely interesting cases, including seven "for diagnosis". These gave rise to a lively discussion. The meeting was well attended and very much enjoyed. The next meeting will be on October 1st at Salisbury. Mr. W. H. Bradbeer has been elected Chairman for 1949–50.

---

BRITISH MEDICAL ASSOCIATION—SOUTH WALES BRANCH

A SUPPER Meeting was held at the Park Hotel, Cardiff, on the evening of 23rd February. Dr. Charles Hill addressed the meeting on "**Current Problems**".

The Annual Dinner of the Society was held at The Royal Hotel, Wednesday, 9th March. Address by Air Marshal P. C. Livingstone, C.B., C.B.E., A.F.C., F.R.C.S., K.H.S., Director-General of Medical Services, R.A.F., at the Engineers' Institute, Park Place, Cardiff; subject: "**Food for Thought**".

---

